# Genetics Meets Metabolomics: A Genome-Wide Association Study of Metabolite Profiles in Human Serum

**DOI:** 10.1371/journal.pgen.1000282

**Published:** 2008-11-28

**Authors:** Christian Gieger, Ludwig Geistlinger, Elisabeth Altmaier, Martin Hrabé de Angelis, Florian Kronenberg, Thomas Meitinger, Hans-Werner Mewes, H.-Erich Wichmann, Klaus M. Weinberger, Jerzy Adamski, Thomas Illig, Karsten Suhre

**Affiliations:** 1 Institute of Epidemiology, Helmholtz Zentrum München, German Research Center for Environmental Health, Neuherberg, Germany; 2 Institute of Medical Informatics, Biometry, and Epidemiology, Ludwig-Maximilians-Universität, Munich, Germany; 3 Institute of Bioinformatics and Systems Biology, Helmholtz Zentrum München, German Research Center for Environmental Health, Neuherberg, Germany; 4 Faculty of Biology, Ludwig-Maximilians-Universität, Planegg-Martinsried, Germany; 5 Institute of Experimental Genetics, Genome Analysis Center, Helmholtz Zentrum München, German Research Center for Environmental Health, Neuherberg, Germany; 6 Institute of Experimental Genetics, Life and Food Science Center Weihenstephan, Technische Universität München, Freising-Weihenstephan, Germany; 7 Division of Genetic Epidemiology, Department of Medical Genetics, Molecular and Clinical Pharmacology, Innsbruck Medical University, Innsbruck, Austria; 8 Institute of Human Genetics, Helmholtz Zentrum München, German Research Center for Environmental Health, Neuherberg, Germany; 9 Institute of Human Genetics, Klinikum rechts der Isar, Technische Universität München, Munich, Germany; 10 Department of Genome-Oriented Bioinformatics, Life and Food Science Center Weihenstephan, Technische Universität München, Freising-Weihenstephan, Germany; 11 Biocrates Life Sciences AG, Innsbruck, Austria; The University of Queensland, Australia

## Abstract

The rapidly evolving field of metabolomics aims at a comprehensive measurement of ideally all endogenous metabolites in a cell or body fluid. It thereby provides a functional readout of the physiological state of the human body. Genetic variants that associate with changes in the homeostasis of key lipids, carbohydrates, or amino acids are not only expected to display much larger effect sizes due to their direct involvement in metabolite conversion modification, but should also provide access to the biochemical context of such variations, in particular when enzyme coding genes are concerned. To test this hypothesis, we conducted what is, to the best of our knowledge, the first GWA study with metabolomics based on the quantitative measurement of 363 metabolites in serum of 284 male participants of the KORA study. We found associations of frequent single nucleotide polymorphisms (SNPs) with considerable differences in the metabolic homeostasis of the human body, explaining up to 12% of the observed variance. Using ratios of certain metabolite concentrations as a proxy for enzymatic activity, up to 28% of the variance can be explained (*p*-values 10^−16^ to 10^−21^). We identified four genetic variants in genes coding for enzymes (FADS1, LIPC, SCAD, MCAD) where the corresponding metabolic phenotype (metabotype) clearly matches the biochemical pathways in which these enzymes are active. Our results suggest that common genetic polymorphisms induce major differentiations in the metabolic make-up of the human population. This may lead to a novel approach to personalized health care based on a combination of genotyping and metabolic characterization. These genetically determined metabotypes may subscribe the risk for a certain medical phenotype, the response to a given drug treatment, or the reaction to a nutritional intervention or environmental challenge.

## Author Summary

This paper reports what is, to the best of our knowledge, the first genome-wide association (GWA) study with metabolic traits as phenotypic traits. By simultaneous measurements of single nucleotide polymorphisms (SNPs) and serum concentrations of endogenous organic compounds in a human population, we identify genetically determined variants in metabolic phenotype (metabotype) that exhibit large effect sizes. Four of these polymorphisms are located in genes coding for well-characterized enzymes of the lipid metabolism. We find that individuals with different genotypes in these genes have significantly different metabolic capacities with respect to the synthesis of some polyunsaturated fatty acids, the beta-oxidation of short- and medium-chain fatty acids, and the breakdown of triglycerides. In this approach, the concept of the “genetically determined metabotype” as an intermediate phenotype is central, as it becomes a measurable quantity in the framework of GWA studies with metabolomics. The investigation of the genetically determined metabotypes in their biochemical context might help to better understand the pathogenesis of common diseases and gene–environment interactions. These findings could result in a step towards personalized health care and nutrition based on a combination of genotyping and metabolic characterization.

## Introduction

**Figure 1:**
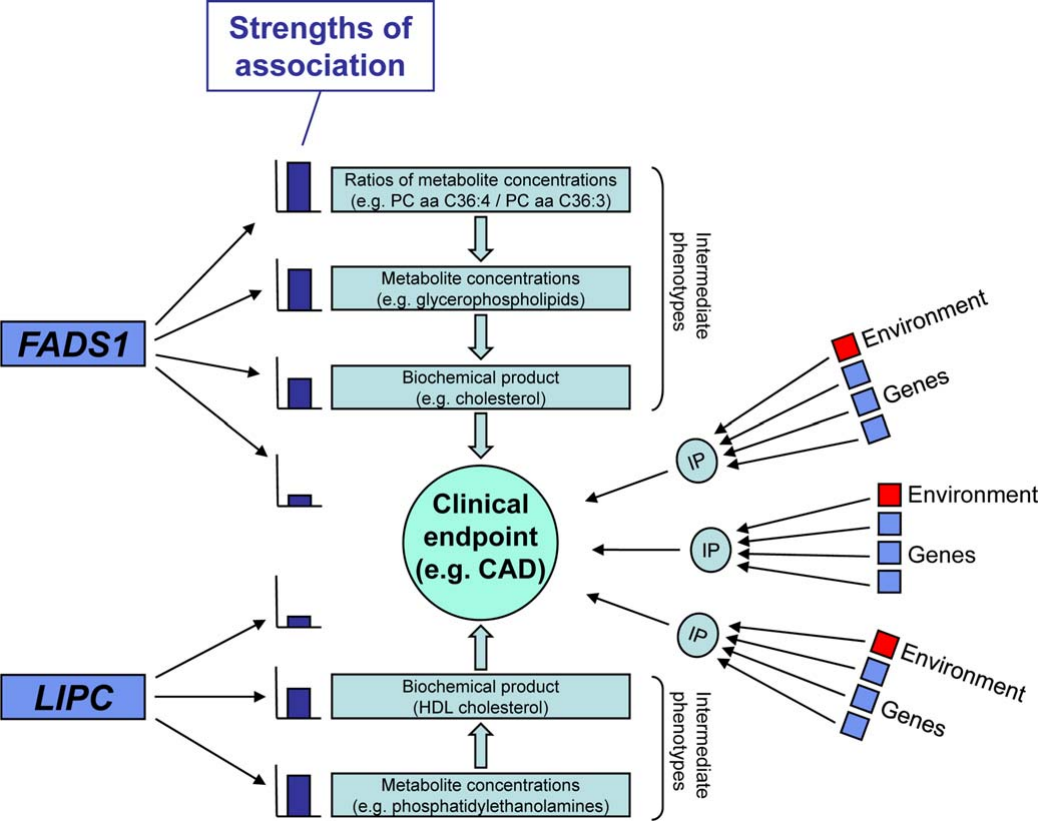
Schematic illustration of the role of intermediate phenotypes (IPs), such as metabolic traits, demonstrated at the examples of two genes that code for major enzymes of the long-chain fatty acid metabolism (*FADS1* and *LIPC*). We show that new information on the functional basis of the observed associations can be inferred from the biochemical properties of the affected metabolites. Moreover, both genes were previously reported to be associated with common clinical phenotypes, *FADS1* in an extent which would not attract immediate attention for follow-up in a genome-wide context. Since several genes and pathways are involved in the development of a clinical endpoint, the IP focuses on one pathway (e.g., cholesterol or a given metabotype) which is already known to be involved in the clinical endpoint (e.g. coronary artery disease (CAD)). It is much easier to identify the genes which are associated with the IP since the associations of genetic variation with the IP is much stronger than with the clinical endpoint. Environmental factors interact at different levels with the IPs and thereby add to the variability in the system. The closer the IP is related to the genetic polymorphism, the stronger the association is expected to be. In our case the association reflects enzymatic activity of *FADS1* and *LIPC* which results in very strong effect sizes of the genetically determined metabotype.

Recent genome-wide association (GWA) studies have identified a number of genetic polymorphisms that convey an increased risk for developing diabetes, coronary artery disease, rheumatoid arthritis, and other common diseases [1]–[4]. However, by only associating genotypes with clinical outcomes, little can be inferred on the disease-causing mechanisms themselves. Moreover, the effect size of genetic associations with clinical phenotypes is often small. Therefore, large populations need to be screened in order to obtain sufficient statistical power for the identification of new disease-causing genetic variants, as recent genome wide association studies with up to 18,000 participants have demonstrated [5]–[7]. Metabolomics, which is the rapidly evolving field of measuring all endogenous metabolites in a cell or body fluid [8]–[16], may contribute to solving this problem. Biochemical measurements of particular intermediate phenotypes on a continuous scale can be expected to provide more details on potentially affected pathways and to be more directly related to the etiology of the disease (Figure 1). It thereby provides a functional readout of the physiological state of the human body. Genetic variants that associate with changes in the homeostasis of key lipids, carbohydrates or amino acids are not only expected to display much larger effect sizes due to their direct involvement in metabolite conversion modification, but may also provide access to the underlying molecular disease-causing mechanisms.

To test this hypothesis, we quantified the concentrations of a comprehensive set of naturally occurring organic compounds from different metabolite classes in blood serum samples from participants of the KORA F3 GWA study [17]–[19], and tested all genotyped common genetic polymorphisms for association with metabolite concentrations as quantitative traits. We show that if the function of the associated gene is known, then the biochemical characteristics of the affected metabolites can support this association and provide information to identify the underlying biological processes. Furthermore, whenever a pair of metabolites is related to the direct substrates and products of an enzymatic conversion, respectively, the ratio between their concentrations can be used as an approximation of the enzymatic activity. We thereby show that the variance in the dataset can be drastically reduced by using these ratios, which increases the power of the GWA study and reduces the corresponding *p*-values of association by several orders of magnitude. Replication of a newly found association in an independent population is the gold standard of all GWA studies. By using metabolite concentrations as proxies for clinical parameters, such as blood cholesterol levels, some of our associations represent replications of previous associations with such parameters.

## Results

**Figure 2:**
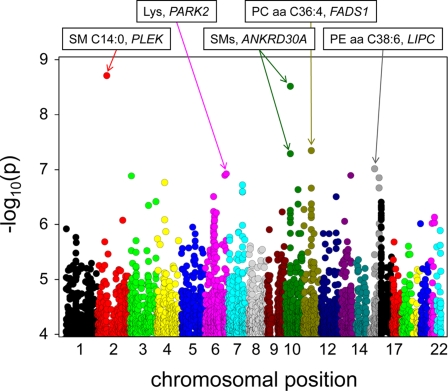
*P*-values of association assuming an additive genetic model, superposing the results obtained from all genome-wide tested metabolic traits. Chromosomal location is indicated by different colors on the x-axis, negative logarithmic *p*-values are reported on the y-axis. The top ranking SNPs together with the closest gene and the most significant associating metabolite(s) are indicated. A complete list of all associations with p<10^−6^ is provided in Table S1, together with significant associations from previous GWA studies with medical phenotypes. Metabolite abbreviations are explained in the material and methods section and a full list of all measured metabolites is provided as supplementary data.

Based on our experience from previous metabolomics studies [20], we chose a targeted quantitative metabolomics platform based on electrospray ionization (ESI) tandem mass spectrometry (MS/MS) to determine the fasting serum concentrations of up to 363 endogenous metabolites, including nine sugar molecules, seven biogenic amines, seven prostaglandins, 29 acylcarnitines, 18 amino acids, 85 sphingolipids, and 208 glycerophospholipids (metabolite naming conventions are defined in the Material and Methods section; a full list of all measured metabolites is available as supplementary material). Data for 201 of these metabolites was obtained for more than 95% of the samples. We conducted a genome-wide association study with these metabolic traits in a group of 284 randomly selected population-based male individuals between 55 and 79 years from the KORA F3 study [18]. Single nucleotide polymorphisms were determined previously on a genome-wide scale for this population using the Affymetrix GeneChip Human Mapping 500K Array Set [17],[19]. To avoid false positive effects from associations based on small numbers, we limited our analysis to SNPs in which at least 5% of the population is homozygous for the minor allele. The corresponding minor allele frequencies are >18.2%. The resulting *p*-values of association for all metabolites when using an additive genetic model are presented in Figure 2. After correction for testing multiple loci and multiple metabolic traits, we estimated a conservative genome-wide level of significance of at least 1.33×10^−9^. None of the associations that we found attained that level when considering isolated metabolic traits. However, the best SNPs rs9309413 (p = 1.95×10^−9^; Table S3) 21 kb upstream of the *PLEK* gene and rs1148259 (p = 3.04×10^−9^; Table S5) in the 3′UTR of *ANKRD30A* were only slightly above genome-wide significance. This is notable because, in contrast to most previous GWA studies, in which association with few and mostly independent phenotypes was tested, a GWA study with metabolomics tests multiple and functionally related outcomes. Moreover, we will show in the following that signals of genome-wide significant levels (*p*-values between 10^−16^ and 10^−21^) can be attained when ratios between metabolite concentrations are used, and that some of our associations can also be considered as true positives on biological grounds.

### A Prototype of a Genetically Determined Metabotype: *FADS1*

**Figure 3:**
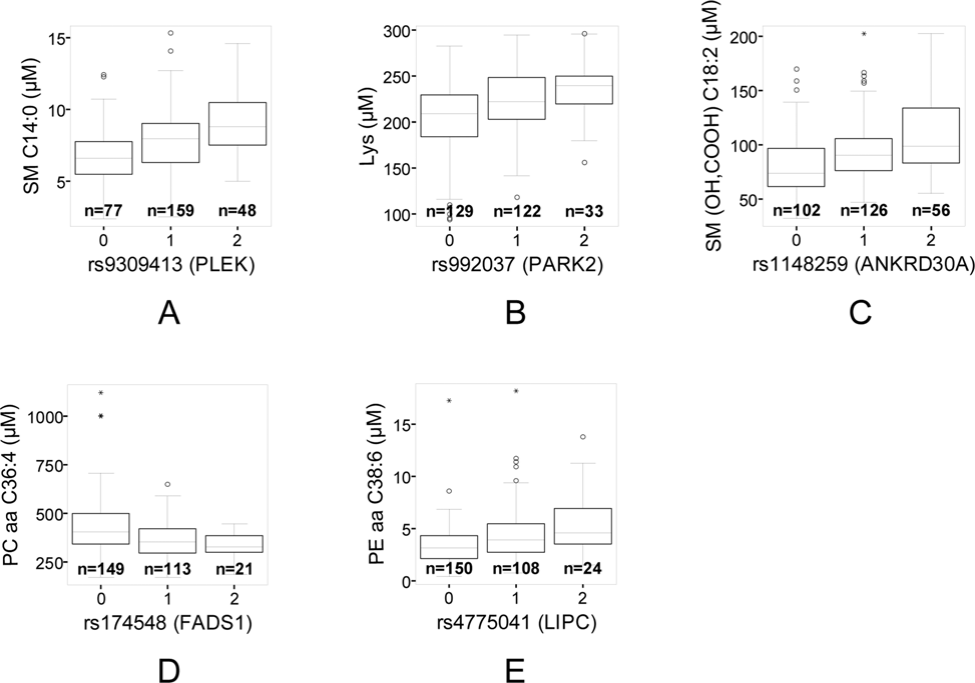
Boxplots of the metabolite concentrations of five top ranking associations as a function of genotype. They show the differentiation of the population that is induced by these genetically determined metabotypes (0 = major allele homozygote, 1 = heterozygote, 2 = minor allele homozygote). Boxes extend from 1^st^ quartile (Q_1_) to 3^rd^ quartile (Q_3_); median is indicated as a horizontal line; whiskers are drawn to the observation that is closest to, but not more than, a distance of 1.5(Q_3_-Q_1_) from the end of the box. Observations that are more distant than this are shown individually on the plot. The number of individuals in each group is given below the boxes. *P*-values for these associations are given in Table 1.

We started our analysis by considering polymorphisms in functionally well characterized enzymes that are among the top ranking association signals in our GWA study (Tables 1 and S1). SNP rs174548, one of several SNPs that lie in a linkage disequilibrium block containing the *FADS1* gene was strongly associated (up to p = 4.52×10^−8^) with a number of glycerophospholipid concentrations (Figure 3 and Table 2). This SNP explains up to 10% of the observed variance of certain glycerophospholipids. The *FADS1* gene codes for the *fatty acid delta-5 desaturase*, a key enzyme in the metabolism of long-chain polyunsaturated omega-3 and omega-6 fatty acids (for a schematic illustration of this pathway see Figure S1). The minor allele variant of this SNP (MAF 27.5%) results in a reduced efficiency of the fatty acid delta-5 desaturase reaction, a fact that can be inferred from the following observations [21],[22]: the concentrations of numerous phosphatidylcholines (PC aa C34:4, PC aa C36:4, PC aa C36:5, PC aa C38:4, PC aa C38:5, PC aa C38:6, PC aa C40:4, PC aa C40:5; metabolite abbreviations are explained in the material and methods section), plasmalogen/plasmenogen phosphatidylcholines (PC ae C36:4, PC ae C38:4, PC ae C38:5, PC ae C38:6, PC ae C40:5), and the phosphatidylinositol PI aa C38:4 with four and more double bonds in their polyunsaturated fatty acid (PUFA) side chains are lowest in individuals that carry the minor allele of rs174548. In particular, the concentrations of the direct product of *FADS1*, arachidonic acid as well as those of its lyso-phosphatidylcholine derivative (PC a C20:4) are found to be significantly reduced with increasing copy number of the minor allele. On the other hand, concentrations of glycerophospholipids with three and less double bonds in their PUFA side chains show a positive association with the *FADS1* genotype. These metabolites include the phosphatidylcholines PC aa C34:2 and PC aa C36:2, the plasmalogen/plasmenogen phosphatidylcholines PC ae C34:2 and PC ae C36:2, the phosphatidylethanolamines PE aa C34:2 and PE aa C36:2, and the phosphatidylinositol PI aa C36:2. The negative association of the sphingomyelin concentrations (SM C22:2, SM C24:2, SM C28:4) can be interpreted as being a result of a changed homeostatis of phosphatidylcholins, since sphingomyelin can be produced from phosphatidylcholine by the action of the sphingomyelin synthase. Similarly, the negative association of the lyso-phosphatidylethanolamin PE a C10:0 can be considered a consequence of the overall changed balance in glycerophospholipid metabolism, since this metabolite can be produced from different phosphatidylethanolamines by abstraction of an arachidonic acid moiety. In summary, we can conclude that the direction of all those associations can be explained by a modification in the efficiency of the fatty acid delta-5 desaturase reaction.

**Table 1:** Genetically determined metabotypes with the strongest signal of association.

Gene	*PLEK*	*PARK2*	*ANKRD30A*	*FADS1*	*LIPC*
Position relative to gene	21 kb upstream	intron	3′UTR	intron	49 kb upstream
rs number	rs9309413	rs992037	rs1148259 (rs1200826)	rs174548	rs4775041
Chromosome	2	6	10	11	15
Chromosomal position	68,482,423	161,971,847	37,548,456	61,327,924	56,461,987
Minor allele frequency	45.2%	34.7%	42.2%	27.5%	28.0%
Best metabolic trait	Sphingomyelin SM C14:0	Lysine	Sphingomyelin SM(OH,COOH) C18:2	Phosphatidylcholine PC aa C36:4	Phosphatidylethanolamine PE aa C38:6
P-value of best metabolic trait	1.95×10^−9^	1.20×10^−7^	3.04×10^−9^	4.52×10^−8^	9.66×10^−8^
Explained variance	12.0%	9.5%	11.7%	10.1%	9.7%
**Traits in GWAS**					
HDL cholesterol^a^	0.035	-	-	1.89×10^−4^	2.80×10^−9^
LDL cholesterol^a^	-	-	-	-	-
Triglycerides^a^	-	-	-	0.0014	7.30×10^−5^
2 h glucose^b^	-	-	-	-	-
2 h insulin^b^	-	-	-	-	-
Apolipoprotein-I, APOA-1^b^	-	-	2.44×10^−4^	0.032	2.75×10^−4^
Apolipoprotein-II, APOA-2^b^	-	-	0.033	0.0055	0.0032
Apolipoprotein B, APOB^b^	-	-	-	-	-
Total cholesterol^b^	-	-	0.043	1.48×10^−4^	0.055
Fasting glucose^b^	-	-	-	-	-
Fasting insulin^b^	-	-	-	-	-
HDL cholesterol^b^	-	-	-	0.037	0.0049
fasting insulin, HOMA^b^	-	-	-	-	-
Insulinogenic index^b^	-	-	-	-	0.016
LDL cholesterol^b^	-	-	0.058	6.07×10^−5^	-
Triglycerides/HDL^b^	0.010	-	-	0.051	0.025
Triglycerides^b^	-	-	-	0.028	0.0071
Bipolar disorder^c^	-	-	-	0.048	0.046
Coronary artery disease^c^	-	-	-	0.021	-
Crohn's disease^c^	-	-	-	0.027	-
Hypertension^c^	-	-	-	-	-
Rheumatoid arthritis^c^	0.031	-	-	-	0.059
Type 1 diabetes mellitus^c^	-	-	-	-	-
Type 2 diabetes mellitus^c^	-	-	-	-	0.061

Reported are the SNP identifier (rs number), chromosome, chromosomal position, the minor allele frequency (MAF), the metabolic trait with the lowest p-value of association (test against the null-hypothesis of no association), and percentage of the variance explained by the additive genetic model. Association results for metabolic traits with p<0.05 are provided in Tables 2, S2, S3, S4, and S5. Data for all 363 metabolic traits are available as supporting online data (Datasets S1 and S2). *P*-values of association from previous GWA studies for the same SNP (neighboring SNP rs1200826 for ANKRD30A) are reported for the following traits: (a) HDL cholesterol, LDL cholesterol, triglycerides are from the publication of Willer et al. [6]; (b) 2 h glucose, 2 h insulin, apolipoproteins A-I, A-II, B, total cholesterol, fasting glucose, fasting insulin, HOMA insulin resistance, insulinogenic index are from the Diabetes Genetics Initiative (DGI) study [5]; (c) bipolar disorder, coronary artery disease, Crohn's disease, hypertension, rheumatoid arthritis, type 1 and type 2 diabetes mellitus are from the WTCCC study [7]. Associations with *p*-values larger than 0.1 are indicated by a ‘-’.

**Table 2:** Associations of rs174548 (*FADS1*) with metabolic traits.

metabolite	mean	ncases	p-value	estimate	explained variance
PC aa C36:4	399.41	284	4.52E-08	−0.318	10.11%
PC a C20:4*	5.09	284	5.30E-07	−0.293	8.58%
PC aa C38:4	209.05	284	4.91E-06	−0.268	7.17%
PC ae C36:5*	19.14	284	1.46E-05	−0.255	6.48%
SM C22:2	4.94	284	5.93E-05	−0.236	5.59%
PC ae C38:4	30.12	284	1.42E-04	−0.224	5.03%
PE aa C34:2	2.22	284	1.54E-04	0.223	4.98%
PC ae C38:5	32.72	284	1.80E-04	−0.221	4.88%
PC aa C38:5	128.89	284	2.01E-04	−0.219	4.81%
PE e (COOH) C16:3*	5.05	284	1.49E-03	0.188	3.53%
PC ae C36:4	35.16	284	1.68E-03	−0.186	3.46%
PE a C10:0	4.16	284	2.34E-03	−0.180	3.25%
PC aa C34:2	810.00	284	2.68E-03	0.178	3.16%
SM (COOH) C18:3	7.30	284	3.08E-03	−0.175	3.07%
PC aa C34:4	3.25	284	3.25E-03	−0.174	3.04%
PC aa C36:5	47.53	284	4.65E-03	−0.168	2.82%
PC ae C36:2	25.33	284	5.87E-03	0.163	2.67%
PC aa C40:5	27.52	284	6.21E-03	−0.162	2.63%
Arachidonic acid	4.33	283	9.04E-03	−0.155	2.41%
PC ae C40:5	6.79	284	1.05E-02	−0.152	2.31%
PC aa C40:4	9.53	284	1.07E-02	−0.151	2.29%
SM (OH) C26:1	12.75	63	1.15E-02	−0.317	10.03%
PI aa C36:2*	7.37	284	1.15E-02	0.150	2.25%
SM C24:2	16.82	221	1.20E-02	−0.169	2.86%
PI aa C38:4*	27.03	284	1.21E-02	−0.149	2.22%
PC aa (OH, COOH) C30:4	342.95	284	1.29E-02	0.148	2.18%
PC ae C34:2	23.44	284	2.28E-02	0.135	1.83%
SM (OH) C24:0	11.80	208	2.94E-02	−0.151	2.29%
LYS	215.17	284	3.05E-02	0.129	1.65%
PA aa C20:7	197.36	284	3.20E-02	−0.128	1.63%
PE aa C36:2	4.42	284	3.52E-02	0.125	1.57%
PC aa (COOH) C30:3*	10.38	215	4.00E-02	0.141	1.98%
PC ae C38:6	11.67	284	4.40E-02	−0.120	1.44%
PC aa C38:6	146.59	284	4.42E-02	−0.120	1.43%
C5-DC	0.11	284	4.43E-02	−0.120	1.43%
SM (OH,COOH) C6:0	4.79	63	4.64E-02	0.252	6.35%
SM C28:4	5.51	284	4.73E-02	−0.118	1.39%
PI a (OH, COOH) C18:2*	3.74	63	4.84E-02	0.250	6.24%
PC aa C36:2	412.59	284	4.92E-02	0.117	1.37%

Metabolites associated (p<0.05) with genotype rs174548 (*FADS1*) in the additive genetic model; in cases where alternative assignments of the metabolites are possible, these are indicated by a ‘*’. Full annotations can be found in the supporting online data files. Reported are the mean concentrations (µM), standard deviation, the number of cases for which metabolite concentrations were obtained (ncases), the p-value of the association, the regression coefficient using an additive genetic model (estimate), and the measure of the observed variance that can be explained by the additive genetic model.

Furthermore, an association of this locus with arachidonic acid and other polyunsaturated fatty acid concentrations has been reported previously in two independent experiments [23],[24]. This case thus constitutes a full replication of this association in a third and independent population, which validates our approach.

### Ratios of Metabolite Concentrations Increase the Power of Association

**Figure 4:**
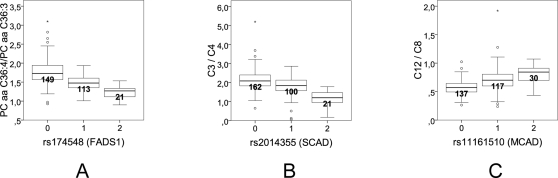
Boxplots of the strongest associations of metabolite concentration ratios with polymorphisms in the FADS1 (A; p = 2.4×10^−22^), SCAD (B; p = 9.3×10^−17^), and MCAD (C; p = 7.6×10^−17^) genes (see legend to Figure 3 for details). The metabolic efficiencies of the reactions that are catalyzed by these three enzymes differ considerably between individuals of different genotype.

We have previously shown that analyzing ratios of metabolite concentrations may strongly reduce the variation in the dataset when a pair of metabolites is closely connected to the direct substrates and products of a given enzymatic reaction [20]. When a tested SNP impacts the efficiency of such a metabolic reaction, using concentration ratios leads to drastically decreased variance, and, consequentially, strongly decreased *p*-values of associations. Such a dependency not only provides rational evidence for a positive association, but also points to potentially affected metabolic pathways, as we demonstrate here for the example of the *FADS1* case. We find that by using metabolite concentration ratios, the p-value of the association with the polymorphism in the *FADS1* gene decreases by up to fourteen orders of magnitude (Table 3). Eicosatrienoyl-CoA (C20:3) and arachidonyl-CoA (C20:4) are the direct substrate and product of the delta-5 desaturase reaction, which is catalyzed by *FADS1*. Synthesis of these metabolites to a glycerol 3-phosphate, and further addition of a palmitoyl-moiety (C16:0), followed by a dephosphorylation step and the addition of a phosphocholin moiety, leads to the formation of the glycerol-phosphatidylcholins PC aa C36:3 and PC aa C36:4, respectively (for a schematic view of the phosphatidylcholine biosynthesis at the example of PC aa C36:4 see Figure S1). PC aa C36:3 and PC aa C36:4 can thus be considered as modified substrates and products of the delta-5 desaturase reaction. If the catalytic activity (or the protein abundance) of *FADS1* is reduced by a polymorphism in its gene (or in a regulatory element), more eicosatrienoyl-CoA (C20:3) and less arachidonyl-CoA (C20:4) is available for the synthesis of those glycerophospholipids that contain these fatty acids. This translates for example into increased PC aa C36:3 concentrations and reduced PC aa C36:4 concentrations. Thus, the ratio between the concentrations of the product-substrate pairs of the delta-5 desaturase reaction, such as [PC aa C36:4]/[ PC aa C36:3] (Figure 4A), will be a strong indicator for the efficiency of the *FADS1* reaction. As reported in Table 3, glycerophospholipids with three double bonds do not associate with the *FADS1* polymorphism (*p*-values ranging from 0.92 to 0.077), whereas the corresponding glycerophospholipids with four double bonds generally display strong associations (most *p*-values ranging from 10^−3^ to 10^−8^). When considering the ratios between concentrations of matched metabolite pairs, the association with the polymorphism in the FADS1 gene increases by up to fourteen orders of magnitude (*p*-values below 10^−21^). This effect is observed not only for one, but for a number of different glycerophospholipid species (PC, PE, PI, incl. plasmalogen/plasmenogen phospholipids) which are thus very likely composed of an arachidonyl-moiety (C20:4) and either a palmitoyl- (C16:0) or a stearoyl-moiety (C18:0), respectively (except for lyso-phosphatidylcholin PC a C20:4, which is formed from a single arachinodyl-moiety). The strongest effect size is observed for phosphatidylcholine diacyl C36:4 (PC aa C36:4) to phosphatidylcholine diacyl C36:3 (PC aa C36:3) ratio (p = 2.4×10^−22^). These metabolites are major constituents of the cell membrane [25 and references therein]. Here, 28.6% of the total variance in the population can be explained by this SNP (Table 3 and Figure 4A). If the molecular function of *FADS1* had not been already known, the association between the SNP and the different glycerophospholipid concentrations per se would have allowed to deduce its enzymatic activity of inserting a fourth double bond into long-chain fatty acids.

**Table 3:** Associations of rs174548 (*FADS1*) with concentrations and ratios between the concentrations of matching pairs of glycerophospholipid species.

enumerator	denominator	mean	ncases	p-value	estimate	explained variance
*Single metabolites (four double bonds)*						
PC a C20:4*	1	5.094	284	5.3×10^−7^	−0.293	8.58%
PC aa C34:4	1	3.249	284	3.3×10^−3^	−0.174	3.04%
PC aa C36:4	1	399.407	284	4.5×10^−8^	−0.318	10.11%
PC aa C38:4	1	209.050	284	4.9×10^−6^	−0.268	7.17%
PC ae C36:4	1	35.160	284	1.7×10^−3^	−0.186	3.46%
PC ae C38:4	1	30.117	284	1.4×10^−4^	−0.224	5.03%
PE aa C38:4	1	5.357	284	0.13	−0.090	0.81%
PI aa C38:4*	1	27.025	284	0.012	−0.149	2.22%
*Single metabolites (three double bonds)*						
PC a C20:3*	1	2.461	208	0.86	−0.013	0.02%
PC aa C34:3	1	30.751	284	0.21	0.075	0.56%
PC aa C36:3	1	250.496	284	0.56	0.035	0.12%
PC aa C38:3	1	123.002	284	0.66	−0.027	0.07%
PC ae C36:3	1	19.697	284	0.17	0.081	0.66%
PC ae C38:3	1	10.641	284	0.74	0.020	0.04%
PE aa C38:3	1	1.623	132	0.92	−0.009	0.01%
PI aa C38:3*	1	7.791	221	0.077	0.120	1.43%
*Ratios between metabolite concentrations*						
PC a C20:4*	PC a C20:3*	2.224	208	2.9×10^−8^	−0.374	13.98%
PC aa C34:4	PC aa C34:3	0.107	284	4.2×10^−7^	−0.295	8.72%
PC aa C36:4	PC aa C36:3	1.613	284	2.4×10^−22^	−0.535	28.62%
PC aa C38:4	PC aa C38:3	1.708	284	2.1×10^−17^	−0.476	22.66%
PC ae C36:4	PC ae C36:3	1.832	284	7.3×10^−8^	−0.313	9.81%
PC ae C38:4	PC ae C38:3	2.888	284	9.7×10^−9^	−0.333	11.07%
PE aa C38:4	PE aa C38:3	3.693	132	0.013	−0.216	4.64%
PI aa C38:4*	PI aa C38:3*	3.582	221	1.5×10^−8^	−0.370	13.69%

Association of SNP rs174548 (*FADS1*) with concentrations and ratios between the concentrations of matching pairs of glycerophospholipid species with three- (denominator) and four-fold (enumerator) unsaturated carbon bonds in their fatty acid side chains; in cases where alternative assignments of the metabolites are possible, these are indicated by a ‘*’; reported are the mean (µM), the number of cases for which metabolite concentrations were obtained (ncases), the p-value of the association, the regression coefficient using an additive genetic model (estimate), and the proportion of the observed variance that can be explained by including the genetic polymorphism in the additive genetic model.

### Association with Medical Phenotypes

Having shown that this polymorphism in the *FADS1* gene strongly influences the serum glycerophospholipid homeostasis, we investigated the effect of this variation on biochemical variables related to medical outcomes. As glycerophospholipids play a major role in cholesterol metabolism, we hypothesized that the *FADS1* polymorphism should have a detectable effect on the corresponding serum parameters when looking at a sufficiently large population. This is indeed the case. Two recent GWA studies with up to 18,000 participants [5],[6] report *p*-values of association for SNP rs174548 with serum low-density lipoprotein (LDL) cholesterol, high-density lipoprotein (HDL) cholesterol and total cholesterol levels that range between 1.89×10^−4^ and 6.07×10^−5^ (Table 1). These associations have not been included into the list of potential candidates for replication in those studies, as their *p*-values taken alone were not sufficiently small in the context of a “classical” GWA study. Our association of SNP rs174548 with different glycerophospholipids can be viewed as an indirect replication of the association of *FADS1* with HDL, LDL and total cholesterol levels in an independent population. Furthermore, we can now hypothesize that the observed change in cholesterol levels induced by this SNP is functionally related to the availability of polyunsaturated long-chain fatty acids with four and more double bonds and its impact on the homeostasis of different glycerophospholipids. This case shows that a combination of a GWA study using metabolomic phenotypic traits with data from previous GWA studies can make it possible to identify promising new candidate SNPs associated to known phenotypes of medical relevance, and to gain new insight into the functional background of these associations.

### A Second Genetically Determined Metabotype that Associates with Medical Phenotype: LIPC

We therefore screened in a further step our strongest associations for overlap with associations in three recent large GWA studies, including serum lipid parameters well known to be involved in cardiovascular diseases as well as seven major common disease phenotypes [5]–[7]. Following this strategy, we identified a series of SNPs in which the biochemical properties of the associated metabolites support the previously reported associations with their clinical outcomes (Tables 1 and S1; Datasets S1 and S2). One example is SNP rs4775041, which is also in the list of our top ranking associations. This SNP is located in a linkage disequilibrium block containing the gene coding for *LIPC*, a key enzyme of the long-chain fatty acid metabolism. This polymorphism associates with the concentrations of numerous glycerophosphatidylcholines, glycerophosphatidylethanolamines and sphingomyelins (up to p = 9.66×10^−8^; Figure 3 and Table S2). For instance, homozygotes carrying the minor allele have on average 70% higher concentrations of the phosphatidylethanolamine diacyl C38:6 (PE aa C38:6) than homozygotes for the major allele. The molecular function of *LIPC* is to break-down triglycerides to diacyl- and monoacylglycerols and fatty acids, which makes this association functionally plausible. In previous GWA studies this locus was reported to be associated with HDL cholesterol (p = 2.80×10^−9^, 3.0×10^−5^, 2.0×10^−3^, and 7.0×10^−3^) and triglyceride levels (p = 7.30×10^−5^) [5],[6],[17],[26].

Our results thus not only replicate the association of *LIPC* with HDL cholesterol and triglyceride levels in an independent population, but, similar to the FADS1 case, they provide new insights into the underlying biochemical mechanism of this association by identifying the involved lipid metabolites. Here we find phosphatidylethanolamines as the most strongly affected metabolites, prompting further research on their role in the cholesterol pathway. For instance, one may speculate that the substrate specificity of *LIPC* is affected by this genetic polymorphism. Interestingly, SNP rs4775041 also weakly associates with type 2 diabetes (p = 0.061), bipolar disorder (p = 0.048) and rheumatoid arthritis (p = 0.059), and this in a third, independent population [7]. These associations are not significant on a genome-wide scale. However, the associations of this polymorphism with phospholipids reported here, as well as its associations with blood cholesterol levels in independent studies suggests that this genetic variant may indeed be causally related to these diseases, albeit further studies in larger populations will be needed to test this hypothesis. In any case, this example indicates how metabolic traits may serve as intermediate phenotypes to identify potential links between genetic variance and complex diseases (see Figure 1).

### Further Examples: PARK2 and PLEK

It is noteworthy that we could identify and validate two associations (*FADS1* and *LIPC*) with major genetically determined metabotypes (concentrations of metabolites and concentration ratios) among the five strongest associations in our GWA study despite the moderate number of participants in this study. We attribute this fact to the unexpectedly large effect sizes in combination with small variances of the genotype-metabotype associations. As it is evident that a number of the other top ranking candidate associations provide information relevant for causal genotype/phenotype associations, we report these results as supplementary material to serve as a resource for further research (Table S1 and Datasets S1 and S2). To give some illustrative examples, a polymorphism in the *PARK2* gene (rs992037; also among the five strongest associations) alters the concentrations of several amino acids. Some of these amino acids are directly connected to the urea cycle (Table S6). *PARK2* codes for parkin, a ubiquitin ligase for which a loss-of-function mutation has been reported to result in Parkinson's disease [27]. When using ratios between metabolite concentrations we observed up to three orders of magnitude smaller *p*-values (Table S6). This suggests that this polymorphism impacts some metabolic pathway that involves glutamate on the one hand and a number of other amino acids (except lysine) on the other hand. Thus, the metabolic footprint of this association is that of an amino acid interconversion, which is supported by the functional role of *PARK2* as a ubiquitin ligase in the protein degradation pathway. Another example for a biologically plausible association is SNP rs9309413, which lies 21 kb upstream of *PLEK*. This SNP has the lowest p-value of association in this study (p = 1.95×10^−9^). The *PLEK* gene codes for pleckstrin, a protein that has been proposed to facilitate protein/lipid interactions and to affect membrane structure [28]. The polymorphism we report here impacts on a number of sphingomyelins, which are known to play a major role in membrane lipid structure (Table S3).

### GWA with Concentration Ratios: SCAD and MCAD

Prompted by the strong increase in the association signal in the FADS1 example by using metabolite concentration ratios, we tested the ratios of all possible metabolite pairs for association with any of the SNPs that have a minor allele frequency higher than 20%. We identified two new loci that are comparable to the FADS1 example in their strength of association and also in terms of the metabolic traits matching the genes' function. The first polymorphism is located in the gene coding for the short-chain acyl-Coenzyme A dehydrogenase (SCAD; e.g. intronic SNP rs2014355, minor allele frequency 25.1%), located on chromosome 12, and the second lies in the gene coding for the medium-chain acyl-Coenzyme A dehydrogenase (MCAD; e.g. intronic SNP rs11161510, minor allele frequency 31.2%) on chromosome 1. Coincidentally, both genes code for enzymes that initiate the beta-oxidation of fatty acids, but they differ in the preference for their chain lengths. The metabolite pair that associates most strongly with rs2014355 of SCAD is the ratio between the short-chain acylcarnitines C3 and C4 (p = 9.3×10^−17^, explained variance 21.8%, Figure 4B) while the pair that associates most strongly with rs11161510 of MCAD is the ratio between the medium-chain acylcarnitines C12 and C8 (p = 7.6×10^−17^, explained variance 21.9%, Figure 4C). Fatty acids are bound to free carnitine for transport and beta-oxidation into the mitochondria. Similar to our argumentation in the FADS1 example, we can therefore consider the short-chain acylcarnitines as indirect substrates and products of SCAD and the medium-chain acylcarnitines as indirect substrates of MCAD, which matches the biochemical function of these enzymes. From the direction of the effect of these polymorphisms (higher concentrations of the longer chain fatty acids ( = substrates) when compared to the smaller chain fatty acids ( = products) implies a reduced dehydrogenase activity) we can further deduce that in both cases minor allele homozygotes have the lowest enzymatic turnover for these reactions.

## Discussion

Our data support the idea that frequent genetically determined metabotypes play a role as discriminating cofactors in the etiology of common multi-factorial diseases. In interactions with environmental factors such as nutrition or life style, these metabotypes may influence the susceptibility of an individual for certain phenotypes. As an example, there is growing evidence (which has yet to be replicated) for a link between the long-chain polyunsaturated fatty acid metabolism and attention deficit/hyperactivity syndrome (ADHS). An association of the same polymorphism in the *FADS1* gene that we identified here (rs174548) has recently been reported to be associated with ADHS [29]. Genetic variation in the *FADS* gene cluster has also been shown to moderate the association between breastfeeding and intelligence quotient (IQ), by influencing the ability to metabolize certain fatty acids that are uniquely available in breast milk [30]. Such effects may possibly be explained by changes in the membrane fluidity of neuronal cells, which depends on the degree of membrane fatty acid saturation, and consequentially impacts the mobility of membrane-bound neuroreceptors.

The differentiation of the population into individuals with different levels of four-fold and higher-fold unsaturated fatty acids, as induced by the *FADS1* polymorphism, is thus a prototype of a genetically determined metabotype. *LIPC*, similar to the case of *FADS1*, corresponds to a second prototypic example of a genetically determined human metabotype. *LIPC* is indeed a factor related to modifications in HDL cholesterol levels, and thereby a cofactor in the etiology of HDL-related diseases, albeit a direct association to such diseases still requires confirmation. Our results clearly demonstrate that a GWA study with metabolomic phenotypes provides a more functional approach to the study of human genetic variation, increases the power of such studies, and allows for the identification or confirmation of new associations from previous GWA studies with clinical parameters as phenotypic traits.

Of particular interest for future research are the two polymorphisms in the SCAD and MCAD genes. Major deficiencies in the corresponding enzymes are known to be associated with severe systemic disorders and with clinical symptoms such as hypoketotic hypoglycemia, lethargy, encephalopathy, and seizures. Such deficiencies are nowadays systematically identified by neonatal screening programs [31 and references therein]. In contrast, the genetic variants that we report here show a relatively moderate phenotypic expression, but are very frequent in the population (minor allele frequencies >25%). One may speculate that individuals that are homozygous for at least one of the minor alleles of the SCAD or MCAD polymorphisms are likely to show signs of impaired beta-oxidation. One would then expect that, for instance in situations of prolonged starvation or physical activity, these individuals may become more readily hypoglycemic and may display the corresponding symptoms, such as tiredness, loss of alertness, headache, and memory problems. It would therefore be promising to search for associations between the SCAD/MCAD polymorphisms and phenotypes that are related to impaired beta-oxidation, possibly in the context of diabetes.

The identification of genetic variants that alter the homeostasis of key metabolites in the human body will eventually lead to a functional understanding of the genetics of complex diseases. To achieve this goal, identifying the major genetically determined metabotypes is mandatory. The current rapid development in the field of metabolomics promises future access to larger metabolite panels, larger population sizes, and metabolomics experiments under different physiological conditions and in different body fluids. This will allow for a more detailed probing of the human metabolic network and its associated genetic variants. We argue that progress towards individualized medication lies in a combination of genotyping and metabotyping, based on evidence provided in part by GWA studies combined with metabolomics like the one presented here. We conclude that metabolomics delivers its promise of providing access to functionally relevant endpoints in the framework of GWA studies, and thereby opens new avenues for a functional investigation of the role of gene-environment interactions in the etiology of complex diseases.

## Material and Methods

### Study Population

This study is based on a previously reported genotyping effort [17],[19] whereof we report the essentials here. We recruited the study population for the genome-wide association study from the KORA S3 survey that is a population-based sample from the general population. The dataset comprises individuals aged 25–74 years resident in the region of Augsburg, Southern Germany, examined in 1994–1995. The standardized examinations applied have been described in detail elsewhere [18 and references therein]. We selected 1,644 subjects, who participated in a follow-up examination of S3 (F3 500K), comprising individuals who, at that time, were aged 35–79 years. With regard to possible effects from population stratification it should be noted that previous work with the KORA F3 500K dataset excluded population stratification as the origin of an observed association with uric acid on the basis of comparison with two other studies [19]. Moreover, possible population stratification in KORA F3 500K was also excluded based on an EIGENSOFT analysis performed in an earlier independent report [32]. Also, recent experimental assessment has found little population stratification to exist within and across Germany [33].

### Genotyping

Genotyping for KORA F3 500K was done using the Affymetrix 500K Array Set, consisting of two chips (*Sty I* and *Nsp I*). Hybridization of genomic DNA was done in accordance with the manufacturer's standard recommendations. Genotypes were determined using the BRLMM clustering algorithm (http://www.affymetrix.com/support/technical/whitepapers/brlmm_whitepaper.pdf). The genotypes were determined in batches of at least 400 chips. For quality control purposes, we applied a positive and a negative control DNA every 48 samples. The overall genotyping efficiency of the GWA was 98.26%. Before statistical analysis, we performed filtering of both conspicuous chips and SNPs based on quality measures to ensure robustness of association analysis. On chip level only subjects with overall genotyping efficiencies of at least 93% for both chips and at most one discordant call for 50 SNPs situated on both chips were included. In addition the called gender has to agree with the gender in the KORA study database. On SNP level from a total of 500,568 SNPs, we excluded for the purpose of this study all SNPs on chromosome×leaving 490,032 autosomal SNPs for the GWA screening step. From these 187,454 SNPs (38.25%) passed all subsequent filter criteria, and were selected for the association analyses presented in this paper. Criteria leading to exclusion were genotyping efficiency <95% (N = 49,325) and genotype frequency of the minor genotype <5% (N = 252,405). An exact Fisher test was used to detect deviations from Hardy Weinberg Equilibrium, and we excluded all SNPs with *p*-values below 10^−6^ (N = 848 after passing the other criteria).

### Sampling

From the 1644 participants genotyped in the KORA F3 500K study population, 284 males (55–79 years) were selected at random for metabolic characterization. Blood samples for metabolic analysis were collected during 2006. To avoid variation due to circadian rhythm, blood was drawn in the morning between 8 and 10 am after a period of overnight fasting. Material was immediately horizontal shaken (10 min), followed by 40 min resting at 4°C to obtain complete coagulation. The material was then centrifuged (2000 g; 4°C). Serum was aliquoted and kept for 2–4 hours at 4°C, after which it was deep frozen to −80°C until sampling.

### Metabolite Measurements

Targeted metabolite profiling by electrospray ionization (ESI) tandem mass spectrometry (MS/MS) was performed on a fee-for-service basis on a quantitative metabolomics platform at *Biocrates Life Sciences AG*, Austria. The company had no access to genotype or phenotype information that would have permitted any data pre-filtering other than objective quality control for measurement errors based on internal controls and duplicates. All metabolomics data was used as received from Biocrates. We did not apply any data correction, nor were any data points removed. The experimental metabolomics measurement technique is described in detail by patent US 2007/0004044 (accessible online at http://www.freepatentsonline.com/20070004044.html). A summary of the method can be found in [34]–[36] and a comprehensive overview of the field and the related technologies is given in the review paper by Wenk [10]. Briefly, a targeted profiling scheme is used to quantitatively screen for known small molecule metabolites using multiple reaction monitoring, neutral loss and precursor ion scans. Quantification of the metabolites of the biological sample is achieved by reference to appropriate internal standards. The method has been proven to be in conformance with 21CFR (Code of Federal Regulations) Part 11, which implies proof of reproducibility within a given error range. It has been applied in different academic and industrial applications [20]. Concentrations of all analyzed metabolites are reported in µM (except for prostaglandin concentrations which are reported in nM units in the supplementary data files).

### Metabolite Panel

In total, 363 different metabolites were detected. The metabolomics dataset contains 18 amino acids, nine reducing mono-, di- and oligosaccharides (abbreviated as H*n* for *n*-hexose, dH for desoxyhexose, UA for uronic acid, HNAc for N-acetylglucosamine, P for Pentose, NANA for N-acetylneuraminic-acid), seven biogenic amines, five prostaglandins, arachidonic acid (AA), docosahexaenoic acid (DHA), free carnitine (C0), 28 acylcarnitines (C*x:y*), hydroxylacylcarnitines (C(OH)*x:y*), and dicarboxylacylcarnitines (C*x:y*-DC), 85 ceramides (Cer), glucosylceramides (GlcCer), different sphingomyelins (SM*x:y*) and sphingomyelin-derivatives, such as N-hydroxyldicarboacyloylsphingosyl-phosphocholine (SM(OH,COOH)*x:y*) and N-hydroxylacyloylsphingosyl-phosphocholine (SM(OH)*x:y*). In addition, 208 phospholipids were detected, including different glycero-phosphatidic acids (PA), glycero-phosphatidylcholines (PC), glycero-phosphatidylethanolamines (PE), phosphatidylglycerols (PG), glycero-phosphatidylinositols (PI), glycero-phosphatidylinositol-bisphosphates (PIP2), and glycero-phosphatidylserines (PS). Glycerophospholipids are further differentiated with respect to the presence of ester (*a*) and ether (*e*) bonds in the glycerol moiety, where two letters (*aa* = diacyl, *ae* = acyl-alkyl, *ee* = dialkyl) denote that two glycerol positions are bound to a fatty acid residue, while a single letter (*a* = acyl or *e* = alkyl) indicates the presence of a single fatty acid residue. Lipid side chain composition is abbreviated as C*x:y*, where *x* denotes the number of carbons in the side chain and *y* the number of double bonds. E.g. “PC ae C33:1” denotes a plasmalogen/plasmenogen phosphatidylcholine with 33 carbons in the two fatty acid side chains and a single double bond in one of them. The precise position of the double bonds and the distribution of the carbon atoms in different fatty acid side chains cannot be determined with this technology. In some cases, the mapping of metabolite names to individual masses can be ambiguous. For example, stereo-chemical differences are not always discernible, neither are isobaric fragments. In such cases, possible alternative assignments are indicated.

### Statistical Analysis

In the statistical analysis only SNPs with a minor allele homozygote frequency of at least 5% were included in order to account for the relatively small sample size of the study. The corresponding smallest minor allele frequency (MAF) in the analyzed dataset is 18.2%. In a first full genome-wide screen, metabolites with less than 5% missing values were used (201 metabolite variables). Additive genetic models assuming a trend per copy of the minor allele were used to specify the dependency of metabolites on genotype categories in the genome wide association study. No further adjustment was performed. The linear regression algorithm implemented in the statistical analysis system R (http://www.r-project.org/) was used in the genome wide association study and SPSS for Windows (Version 15.0, Chicago: SPSS Inc.) was used for statistical analysis on a case-by-case level. It should be noted that the calculation of p values is based on asymptotic assumptions, which do not apply down to extremely low levels. Such *p*-values should thus be interpreted merely as indicators for the strength of an association, but not as absolute probabilities. A conservative estimate of a genome-wide significance level (using the Bonferroni correction) based on a nominal level of 0.05 is 1.33×10^−9^ (0.05 / (201*187,454) ). However, such a small p-value of an association would only be required to confirm an association between a SNP and a single metabolite concentration if all SNPs and metabolites were acting independently. As explained in the main text, in the case of a GWA study with metabolomics, evidence from multiple metabolic traits can be combined into a multi-factorial “metabolic story”, where changes in metabolite concentrations are interpreted in the context of their position on the metabolic pathways. To document the complete story all SNPs that associate with at least one metabolic trait, associations with a p-value smaller than 10^−4^ are retained for further analysis (2927 SNPs). For these cases, all other metabolic traits that also associate with the same SNP with a p-value of association smaller than 0.01, are reported and made available as supporting online data (30641 associations) (Datasets S1 and S2). These have been selected from a set of 187,454 SNPs. Moreover, the metabolic measures are not independent, and therefore if by chance one trait associates with an SNP its correlate would also be expected to associate with that SNP. For the top ranking associations we then carried out a linear regression between the associating SNP (additive genetic model), using all available (max. 363) metabolite concentrations as quantitative traits. In addition, motivated by our previous observation [20] that the use of ratios may lead to a strong reduction in the overall variance and a corresponding improvement in the *p*-values of association, we computed all possible pairs of metabolite concentration ratios for those cases and used those ratios as quantitative traits in a subsequent test. A strong reduction in p-value indicates that two metabolites may be linked by a metabolic pathway that is modified by the SNP. A conservative estimation of the genome-wide significance level (Bonferroni correction) when testing all metabolite pairs, based on a nominal level of 0.05, is 6.6×10^−12^ (1.33×10^−9^/ 201). The results of these computations are provided as supporting online data for the cases discussed in this paper. It should be noted that we report there some particular genetic variants down to p = 0.05.

### GWA Data from Other Studies

Genome wide association data (*p*-values) from three recent GWA studies was downloaded on 21 February 2008 from http://www.broad.mit.edu/diabetes/scandinavs/metatraits.html (Broad Institute [5]) and http://www.sph.umich.edu/csg/abecasis/public/lipids/ (University of Michigan [6]) and on 14 March 2008 from http://www.wtccc.org.uk/info/summary_stats.shtml (Wellcome Trust Case Control Consortium [7]). All *p*-values of association of these three GWA studies were combined with our dataset. In the WTCCC study several methods to compute *p*-values of association were used. Here we only use *p*-values using the additive frequentist model on the base population (controls and suitable cases merged as described in [7]). Data points from that study that were flagged as having bad clustering parameters in the genotype calling were excluded.

## Supporting Information

Figure S1 Schematic view of the role of FADS1 in the synthesis of phosphatidylcholine. Long-chain poly-unsaturated fatty acids have to be produced from the essential fatty acid linoleic acids (C18:2) in the omega-6 fatty acid synthesis pathway (top figure) and from alpha-linolenic acid (C18:3) in the omega-3 fatty acid synthesis pathway (not shown). Un- and monosaturated fatty acids with chain lengths of up to 18 carbons, i.e. palmic acid (C16:0), stearic acid (C18:0) and oleic acid (C18:1) can be synthesized de novo in the human body. In the Kennedy pathway, glycerol-phosphatidylcholins (PC) with different fatty acid side chains are then produced from two fatty acid moieties (bottom figure). These are linked to a glycerol 3-phosphate, followed by a dephosphorylation step and the addition of a phosphocholin moiety. A very good review of the underlying lipid metabolism can be found in Vance (2001). Figures and pathways shown here were adapted from the KEGG database at http://www.genome.jp/kegg/ (Kanehisa et al. 2006).
(0.53 MB TIF)

Table S1 List of top ranking associations. List of all associations with a p-value of association smaller than 10^−6^ for at least one of the tested metabolic traits. Reported are the SNP identifier (rs number), its chromosome (Chr.) and its chromosomal position (Position), the minor allele frequency (MAF), and the metabolic trait with the lowest p-value of association (test against the null-hypothesis of no association); where an association (p<0.1) of the same SNP has been reported in one of the recent GWA studies (WTCCC 2007; Kathiresan et al. 2008; Willer et al. 2008), the p-value of the strongest association is reported in the comment column. Abbreviations are explained in the legend to Table 1. More details and associations up to p<10^−4^ are provided in supporting online Dataset S1.
(0.08 MB DOC)

Table S2 Associations of rs4775041 (LIPC) with metabolic traits. Metabolites associated (p<0.05) with genotype rs4775041 (LIPC) in the additive genetic model. In cases where alternative assignments of the metabolites are possible, these are indicated by a ‘*’. Full annotations can be found in the supporting online data files. Reported are the mean concentrations (µM), standard deviation, the number of cases for which metabolite concentrations were obtained (ncases), the p-value of the association, the regression coefficient using an additive genetic model (estimate), and the measure of the observed variance that can be explained by the additive genetic model.
(0.08 MB DOC)

Table S3 Associations of rs9309413 (PLEK) with metabolic traits. Metabolites associated (p<0.05) with genotype rs9309413 (PLEK) in the additive genetic model (see Table S2 for legend).
(0.09 MB DOC)

Table S4 Associations of rs1148259 (ANKRD30A) with metabolic traits. Metabolites associated (p<0.05) with genotype rs1148259 (ANKRD30A) in the additive genetic model (see Table S2 for legend).
(0.09 MB DOC)

Table S5 Associations of rs992037 (PARK2) with metabolic traits. Metabolites associated (p<0.05) with genotype rs992037 (PARK2) in the additive genetic model (see Table S2 for legend).
(0.08 MB DOC)

Table S6 Associations of rs992037 (PARK2) with metabolite concentration ratios. Selected metabolite concentration ratios associated (p<0.05) with genotype rs992037 (PARK2) in the additive genetic model (see Table S2 for legend; ncases = 284). The improvement of the p-value of association when using metabolite concentration ratios is calculated based on the following formula: min(p[C_enumerator], p[C_nominator]) / p[C_enumerator / C_nominator], where C_ is a metabolite concentration and p[.] the corresponding p-value of association.
(0.06 MB DOC)

Dataset S1 Association data for all associations with p<10^−4^: EXCEL worksheet reporting all SNPs (minor allele homozygote frequency >5%) that associate with at least one metabolic trait (2927 SNPs); all other metabolic traits that also associate with the same SNP with a p-value of association smaller than 0.01 are also reported (30641 associations), together with the corresponding p-values from previous GWA studies (as described in the manuscript).
(4.48 MB ZIP)

Dataset S2 List of all measured metabolites and their biochemical classification; complete association dataset for the top ranking cases: EXCEL worksheets comprising the regression results of all 363 metabolites against the genotypes listed in Table 1 in the additive genetic model (sheets PLEK, ANKRD30A, LIPC, FADS1, PARK2, SCAD, MCAD) and EXCEL worksheets comprising the regression results of all 363×363 metabolite concentration ratios against the genotypes listed in Table 1 in the additive genetic model, limited to metabolite pairs with p-values <10^−4^ when using metabolite concentration ratios (sheets PLEK_ratios, ANKRD30A_ratios, LIPC_ratios, FADS1_ratios, PARK2_ratios, SCAD_ratios, MCAD_ratios).
(0.40 MB ZIP)
