# Supplementary figures and images for: Genetics Meets Metabolomics: A Genome-Wide Association Study of Metabolite Profiles in Human Serum
Source: PLoS Genet. 2008 Nov 28;4(11):e1000282. doi: 10.1371/journal.pgen.1000282 (PMC2581785; doi:10.1371/journal.pgen.1000282)

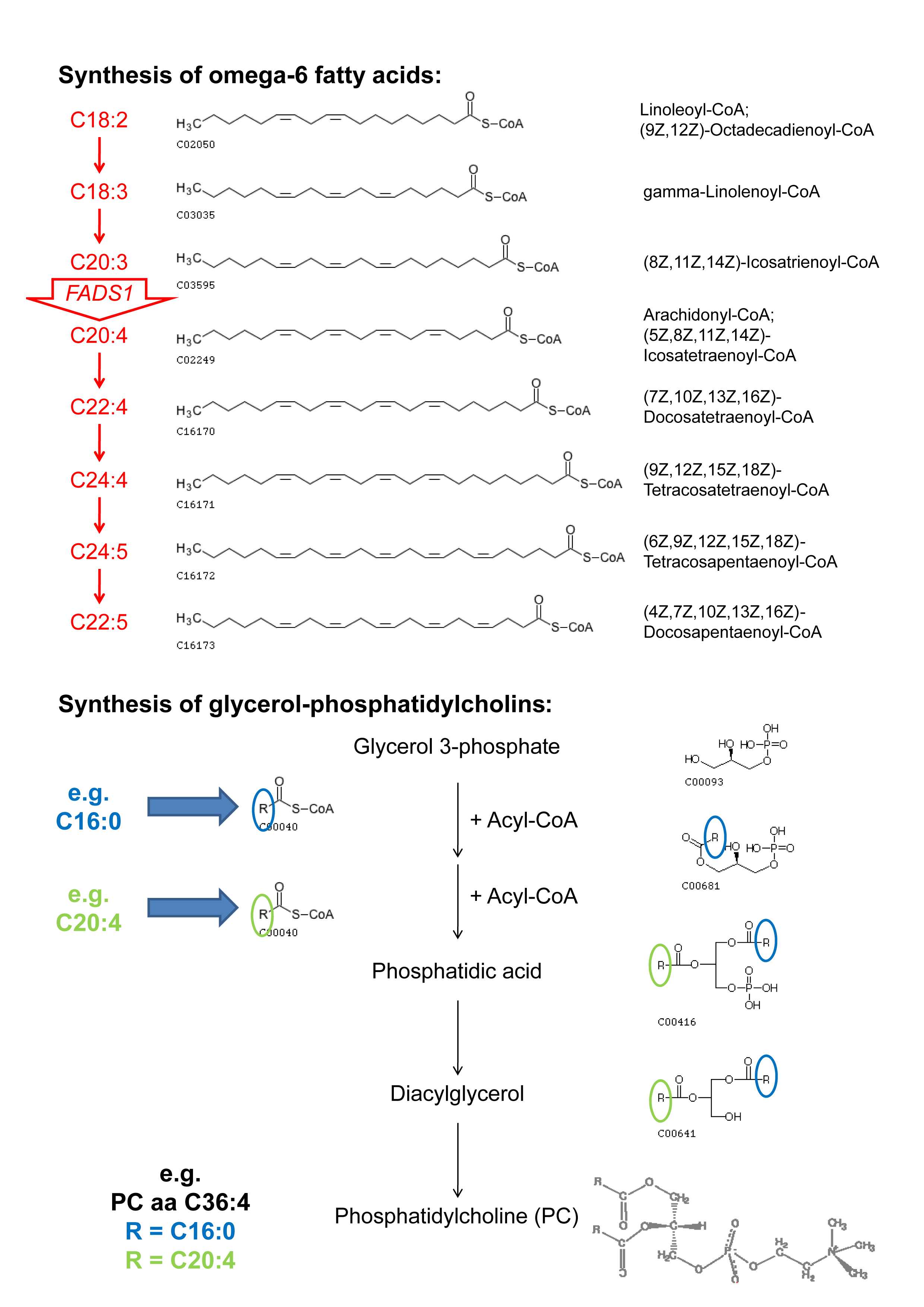

Supplement: Figure S1 — Schematic view of the role of FADS1 in the synthesis of phosphatidylcholine. Long-chain poly-unsaturated fatty acids have to be produced from the essential fatty acid linoleic acids (C18:2) in the omega-6 fatty acid synthesis pathway (top figure) and from alpha-linolenic acid (C18:3) in the omega-3 fatty acid synthesis pathway (not shown). Un- and monosaturated fatty acids with chain lengths of up to 18 carbons, i.e. palmic acid (C16:0), stearic acid (C18:0) and oleic acid (C18:1) can be synthesized de novo in the human body. In the Kennedy pathway, glycerol-phosphatidylcholins (PC) with different fatty acid side chains are then produced from two fatty acid moieties (bottom figure). These are linked to a glycerol 3-phosphate, followed by a dephosphorylation step and the addition of a phosphocholin moiety. A very good review of the underlying lipid metabolism can be found in Vance (2001). Figures and pathways shown here were adapted from the KEGG database at http://www.genome.jp/kegg/ (Kanehisa et al. 2006). (0.53 MB TIF) [file pgen.1000282.s001.tif]
