# Supplementary material for: Genetics Meets Metabolomics: A Genome-Wide Association Study of Metabolite Profiles in Human Serum
Source: PLoS Genet. 2008 Nov 28;4(11):e1000282. doi: 10.1371/journal.pgen.1000282 (PMC2581785; doi:10.1371/journal.pgen.1000282)
Supplement: Table S1 — List of top ranking associations. List of all associations with a p-value of association smaller than 10−6 for at least one of the tested metabolic traits. Reported are the SNP identifier (rs number), its chromosome (Chr.) and its chromosomal position (Position), the minor allele frequency (MAF), and the metabolic trait with the lowest p-value of association (test against the null-hypothesis of no association); where an association (p<0.1) of the same SNP has been reported in one of the recent GWA studies (WTCCC 2007; Kathiresan et al. 2008; Willer et al. 2008), the p-value of the strongest association is reported in the comment column. Abbreviations are explained in the legend to Table 1. More details and associations up to p<10−4 are provided in supporting online Dataset S1. (0.08 MB DOC) [file pgen.1000282.s002.doc]

| **rs number** | **Chr.** | **Position** | **MAF** | **best metabolic trait** | **p-value** | **Comment** |
| --- | --- | --- | --- | --- | --- | --- |
| rs9309413 | 2 | 68,482,423 | 45.2% | Sphingomyelin  SM C14:0 | 1.95E-9 | 21kb upstream PLEK (pleckstrin);  triglyceride/HDL (p=0.010) |
| rs6807064 | 3 | 10,510,771 | 29.3% | Phenylalanine | 1.31E-07 | 44kb upstream ATP2B2 (ATPase, plasma membrane calcium pump);  TG (p=0.023) |
| rs1382269 | 3 | 138,591,180 | 47.0% | Sphingomyelin  SM (OH,COOH) C20:3 | 4.52E-07 | intergenic;  LDL (p=0.078) |
| rs4453795 | 3 | 193,576,677 | 40.9% | Phosphatidylcholine  PC aa C34:4 | 3.86E-07 | intron FGF12  (fibroblast growth factor 12);  BD (p=0.059) |
| rs10517480 | 4 | 60,577,595 | 30.6% | Phosphatidylcholine  PC ae C38:3 | 1.74E-07 | intergenic |
| rs9354308 | 6 | 66,622,074 | 36.7% | Serotonin | 3.12E-07 | intergenic;  RA (p=0.044) |
| rs9342503 | 6 | 66,622,157 | 36.7% | Serotonin | 3.12E-07 | intergenic;  RA (p=0.049) |
| rs9360161 | 6 | 66,622,178 | 36.7% | Serotonin | 3.12E-07 | intergenic;  RA (p=0.040) |
| rs1591830 | 6 | 150,744,014 | 32.3% | Sugar  H3-HNAc2-NANA | 1.28E-07 | 39kb upstream C6orf71  (iodotyrosine deiodinase);  TG (p=0.0192) |
| rs992037 | 6 | 161,971,847 | 34.7% | Lysine | 1.20E-07 | intron PARK2 (parkin) |
| rs10953730 | 7 | 112,505,361 | 34.8% | Acylcarnitine  C12  C4:1-DC  C10 | 1.93E-07  2.54E-07  2.58E-07 | intergenic;  T2D (p=0.0072) |
| rs1148259 | 10 | 37,548,456 | 42.2% | Sphingomyelin  SM(OH,COOH) C18:2 | 3.04E-09 | 3'UTR ANKRD30A  (ankyrin repeat domain 30A) |
| rs1200826 | 10 | 37,604,993 | 48.2% | Sphingomyelin  SM(OH,COOH) C18:2 | 5.14E-08 | 43 kb downstream ANKRD30A  (ankyrin repeat domain 30A);  APOA1 (p=2.44x10^-4^) |
| rs7081443 | 10 | 94,997,483 | 47.2% | Phosphatidylethanoamine  PE a C10:0 | 4.27E-07 | 59kb downstream FER1L3  (myoferlin);  TG (p=0.025) |
| rs12765326 | 10 | 95,004,928 | 47.2% | Phosphatidylethanoamine  PE a C10:0 | 2.34E-07 | intergenic;  TG (p=0.028) |
| rs174548 | 11 | 61,327,924 | 27.5% | Phosphatidylcholine  PC aa C36:4 | 4.52E-08 | intron FADS1  (fatty acid desaturase 1);  LDL (p=6.07x10^-5^) |
| rs174549 | 11 | 61,327,958 | 27.0% | Phosphatidylcholine  PC aa C36:4 | 2.20E-07 | intron FADS1  (fatty acid desaturase 1);  LDL (p=9.22x10^-5^) |
| rs174455 | 11 | 61,412,693 | 35.9% | Phosphatidylethanoamine  PE a C18:2 | 4.23E-07 | intron FADS3  (fatty acid desaturase 3);  HDL (p=8.13x10^-5^) |
| rs2194980 | 12 | 113,965,438 | 33.2% | Tyrosine | 3.14E-07 | intergenic;  2h glucose (p=0.051) |
| rs17267292 | 13 | 92,121,147 | 27.7% | Docosahexaonic acid | 1.28E-07 | intron GPC5 (glypican 5);  residual cholesterol (p=0.017) |
| rs4775041 | 15 | 56,461,987 | 28.0% | Phosphatidylethanoamine  PE aa C38:6 | 9.66E-08 | 49kb upstream LIPC  (hepatic lipase);  HDL (p=2.80x10^-9^) |
| rs756873 | 15 | 89,518,332 | 37.6% | Sphingomyelin  SM (COOH) C18:3 | 1.42E-07 | 52kb upstream SV2B  (synaptic vesicle glycoprotein 2B);  APOB (p=0.052) |
| rs886144 | 15 | 89,518,356 | 37.6% | Sphingomyelin  SM (COOH) C18:3 | 2.17E-07 | 52kb upstream SV2B  (synaptic vesicle glycoprotein 2B);  T1D (p=0.065) |
| rs9935875 | 16 | 7,444,597 | 41.2% | Phosphatidylcholine  PC ae C34:2 | 4.57E-07 | intron A2BP1  (ataxin 2-binding protein 1) |
| rs9935962 | 16 | 7,444,671 | 41.3% | Phosphatidylcholine  PC ae C34:2 | 3.93E-07 | intron A2BP1  (ataxin 2-binding protein 1) |
| rs9924951 | 16 | 7,444,855 | 40.6% | Phosphatidylcholine  PC ae C34:2 | 4.97E-07 | intron A2BP1  (ataxin 2-binding protein 1) |
| rs9936248 | 16 | 7,445,030 | 41.5% | Phosphatidylcholine  PC ae C34:2 | 4.70E-07 | intron A2BP1  (ataxin 2-binding protein 1);  HDL (p=0.074) |
