# Supplementary material for: Genetics Meets Metabolomics: A Genome-Wide Association Study of Metabolite Profiles in Human Serum
Source: PLoS Genet. 2008 Nov 28;4(11):e1000282. doi: 10.1371/journal.pgen.1000282 (PMC2581785; doi:10.1371/journal.pgen.1000282)
Supplement: Table S2 — Associations of rs4775041 (LIPC) with metabolic traits. Metabolites associated (p<0.05) with genotype rs4775041 (LIPC) in the additive genetic model. In cases where alternative assignments of the metabolites are possible, these are indicated by a ‘*’. Full annotations can be found in the supporting online data files. Reported are the mean concentrations (µM), standard deviation, the number of cases for which metabolite concentrations were obtained (ncases), the p-value of the association, the regression coefficient using an additive genetic model (estimate), and the measure of the observed variance that can be explained by the additive genetic model. (0.08 MB DOC) [file pgen.1000282.s003.doc]

| **Metabolite** | **mean** | **ncases** | **p-value** | **estimate** | **explained variance** |
| --- | --- | --- | --- | --- | --- |
| PE aa C38:6 | 3.921 | 284 | 9.7E-08 | 0.311 | 9.67% |
| PE aa C40:6 | 3.455 | 284 | 7.9E-06 | 0.263 | 6.89% |
| PE aa C38:5 | 3.024 | 284 | 4.2E-04 | 0.209 | 4.36% |
| PE aa C36:4 | 2.731 | 284 | 7.5E-04 | 0.200 | 3.98% |
| PC aa (COOH) C30:4 | 4.870 | 76 | 2.1E-03 | 0.347 | 12.04% |
| PE aa C38:4 | 5.357 | 284 | 3.2E-03 | 0.175 | 3.06% |
| PE aa C34:2 | 2.221 | 284 | 4.0E-03 | 0.171 | 2.93% |
| PC aa (COOH) C30:3* | 10.379 | 215 | 4.9E-03 | 0.192 | 3.68% |
| SM C16:0 | 77.224 | 284 | 1.4E-02 | 0.146 | 2.14% |
| PC ae C34:6* | 2.852 | 284 | 1.4E-02 | 0.145 | 2.12% |
| PE aa C40:5 | 1.575 | 132 | 1.5E-02 | 0.211 | 4.43% |
| TRP | 79.774 | 284 | 1.8E-02 | -0.140 | 1.97% |
| SM C16:1 | 9.982 | 284 | 1.8E-02 | 0.140 | 1.97% |
| SM C28:3 | 2.592 | 284 | 1.9E-02 | 0.139 | 1.94% |
| PC aa C38:6 | 146.588 | 284 | 2.1E-02 | 0.138 | 1.89% |
| SM (COOH) C16:2 | 2.926 | 284 | 2.3E-02 | 0.136 | 1.84% |
| SM C14:0 | 7.787 | 284 | 2.4E-02 | 0.135 | 1.81% |
| XLEU | 136.470 | 284 | 3.0E-02 | -0.129 | 1.67% |
| SM (OH,COOH) C14:1 | 2.162 | 221 | 3.1E-02 | 0.146 | 2.13% |
| PC ae C40:5 | 6.790 | 284 | 3.3E-02 | 0.127 | 1.62% |
| PI a (OH) C12:3 | 4.063 | 139 | 3.4E-02 | 0.181 | 3.28% |
| PE aa C36:2 | 4.422 | 284 | 3.4E-02 | 0.126 | 1.59% |
| VAL | 177.570 | 284 | 3.9E-02 | -0.123 | 1.51% |
| PC aa (COOH) C30:2 | 10.572 | 215 | 4.1E-02 | 0.140 | 1.97% |
| PC ae C38:6 | 11.669 | 284 | 4.1E-02 | 0.122 | 1.48% |
| PC ae (OH, COOH) C30:3 | 10.582 | 215 | 4.1E-02 | 0.140 | 1.96% |
| PC aa C40:6 | 46.067 | 284 | 4.4E-02 | 0.120 | 1.44% |
| PI aa C38:3* | 7.791 | 221 | 4.5E-02 | 0.136 | 1.84% |
| PE a (OH, COOH) C12:2 | 2.864 | 284 | 4.6E-02 | 0.119 | 1.41% |
| PE e (OH) C18:1 | 3.297 | 63 | 4.9E-02 | 0.249 | 6.22% |
| PC aa (COOH) C26:2* | 3.711 | 152 | 4.9E-02 | 0.161 | 2.60% |
| GLU | 129.766 | 284 | 4.9E-02 | -0.117 | 1.37% |
