# Supplementary material for: Genetics Meets Metabolomics: A Genome-Wide Association Study of Metabolite Profiles in Human Serum
Source: PLoS Genet. 2008 Nov 28;4(11):e1000282. doi: 10.1371/journal.pgen.1000282 (PMC2581785; doi:10.1371/journal.pgen.1000282)
Supplement: Table S3 — Associations of rs9309413 (PLEK) with metabolic traits. Metabolites associated (p<0.05) with genotype rs9309413 (PLEK) in the additive genetic model (see Table S2 for legend). (0.09 MB DOC) [file pgen.1000282.s004.doc]

| **metabolite** | **mean** | **ncases** | **p-value** | **estimate** | **explained variance** |
| --- | --- | --- | --- | --- | --- |
| SM C14:0 | 7.79 | 284 | 1.95E-09 | 0.347 | 12.01% |
| SM C16:0 | 77.22 | 284 | 4.34E-04 | 0.207 | 4.30% |
| PC ae C42:3 | 1.56 | 132 | 1.31E-03 | 0.277 | 7.67% |
| SM C22:1 | 13.76 | 284 | 1.95E-03 | 0.183 | 3.35% |
| SM (COOH) C12:0 | 0.96 | 208 | 1.97E-03 | 0.213 | 4.55% |
| SM C18:0* | 16.46 | 284 | 3.35E-03 | 0.174 | 3.01% |
| SM C26:4 | 3.51 | 284 | 3.94E-03 | 0.171 | 2.91% |
| PC aa (COOH) C26:2* | 3.71 | 152 | 5.69E-03 | 0.223 | 4.99% |
| SM C24:4 | 118.12 | 60 | 6.42E-03 | -0.348 | 12.12% |
| SM C24:3 | 3.89 | 284 | 7.47E-03 | 0.158 | 2.51% |
| PC aa C28:1* | 4.72 | 69 | 7.48E-03 | 0.319 | 10.20% |
| PC aa (OH, COOH) C28:4 | 6.61 | 284 | 7.59E-03 | 0.158 | 2.50% |
| PI aa (OH, COOH) C28:2 | 24.13 | 139 | 7.80E-03 | -0.225 | 5.05% |
| SM (COOH) C18:1 | 7.84 | 284 | 1.02E-02 | 0.152 | 2.32% |
| SM C18:1 | 7.47 | 284 | 1.07E-02 | 0.151 | 2.29% |
| SM C24:2 | 16.82 | 221 | 1.11E-02 | 0.171 | 2.91% |
| PC ae C38:1* | 11.49 | 284 | 1.21E-02 | 0.149 | 2.21% |
| PE aa (COOH) C14:1 | 1.40 | 63 | 1.26E-02 | 0.313 | 9.78% |
| PE ae (OH, COOH) C14:2 | 1.43 | 63 | 1.27E-02 | 0.312 | 9.76% |
| PC ae C40:5 | 6.79 | 284 | 1.33E-02 | 0.147 | 2.15% |
| SM C22:3 | 2.99 | 208 | 1.34E-02 | 0.171 | 2.93% |
| SM C20:4 | 206.98 | 60 | 1.52E-02 | -0.312 | 9.74% |
| PC ae C40:4 | 4.81 | 284 | 1.83E-02 | 0.140 | 1.96% |
| PC ae C38:0* | 5.24 | 284 | 1.98E-02 | 0.138 | 1.91% |
| SM C20:3 | 10.73 | 284 | 2.18E-02 | 0.136 | 1.85% |
| SM (OH) C20:1 | 16.74 | 284 | 2.58E-02 | 0.132 | 1.75% |
| PC ae C36:2 | 25.33 | 284 | 2.59E-02 | 0.132 | 1.75% |
| PC ae C32:7* | 10.96 | 221 | 2.60E-02 | 0.150 | 2.24% |
| SM C20:1 | 4.18 | 284 | 2.61E-02 | 0.132 | 1.74% |
| PE e C14:0 | 82.97 | 132 | 2.75E-02 | 0.192 | 3.68% |
| PC aa (COOH) C30:3 | 10.38 | 215 | 2.79E-02 | 0.150 | 2.25% |
| PC ae (OH, COOH) C30:4 | 10.38 | 215 | 2.79E-02 | 0.150 | 2.25% |
| Docosahexaonic acid | 4.14 | 283 | 3.31E-02 | 0.127 | 1.61% |
| PC aa C36:2 | 412.59 | 284 | 3.41E-02 | 0.126 | 1.58% |
| SM C26:3 | 3.28 | 284 | 3.55E-02 | 0.125 | 1.56% |
| PC aa (OH, COOH) C30:4 | 342.95 | 284 | 3.70E-02 | 0.124 | 1.53% |
| SM (COOH) C16:0 | 3.31 | 284 | 4.06E-02 | 0.122 | 1.48% |
| PC ae C32:0* | 7.89 | 284 | 4.67E-02 | 0.118 | 1.40% |
| PC aa (COOH) C14:2 | 5.22 | 215 | 4.70E-02 | 0.136 | 1.84% |
| SM C22:0 | 21.51 | 221 | 4.77E-02 | 0.133 | 1.78% |
| PC ae (COOH) C30:3 | 7.37 | 208 | 4.80E-02 | 0.137 | 1.88% |
| PC a C18:1* | 15.55 | 284 | 4.92E-02 | 0.117 | 1.36% |
