# Supplementary material for: Genetics Meets Metabolomics: A Genome-Wide Association Study of Metabolite Profiles in Human Serum
Source: PLoS Genet. 2008 Nov 28;4(11):e1000282. doi: 10.1371/journal.pgen.1000282 (PMC2581785; doi:10.1371/journal.pgen.1000282)
Supplement: Table S4 — Associations of rs1148259 (ANKRD30A) with metabolic traits. Metabolites associated (p<0.05) with genotype rs1148259 (ANKRD30A) in the additive genetic model (see Table S2 for legend). (0.09 MB DOC) [file pgen.1000282.s005.doc]

| **metabolite** | **mean** | **ncases** | **p-value** | **estimate** | **explained variance** |
| --- | --- | --- | --- | --- | --- |
| SM (OH,COOH) C18:2 | 92.45 | 284 | 3.04E-09 | 0.343 | 11.74% |
| SM (OH,COOH) C16:1 | 27.23 | 284 | 6.40E-07 | 0.290 | 8.43% |
| SM (OH,COOH) C18:1 | 19.27 | 284 | 7.81E-07 | 0.288 | 8.30% |
| SM (OH,COOH) C16:2* | 84.94 | 284 | 1.46E-06 | 0.281 | 7.91% |
| SM (OH,COOH) C22:0 | 17.32 | 284 | 2.67E-06 | 0.274 | 7.53% |
| SM (OH) C22:1 | 44.57 | 284 | 5.59E-06 | 0.266 | 7.06% |
| SM (OH,COOH) C20:2 | 64.74 | 284 | 7.61E-06 | 0.262 | 6.87% |
| SM (OH) C20:3 | 172.87 | 284 | 2.30E-05 | 0.248 | 6.17% |
| SM (OH,COOH) C20:4 | 74.88 | 284 | 3.13E-05 | 0.244 | 5.97% |
| SM (OH,COOH) C24:0 | 20.93 | 284 | 5.78E-05 | 0.236 | 5.58% |
| SM (OH,COOH) C16:0 | 56.42 | 284 | 6.17E-05 | 0.235 | 5.54% |
| SM (OH,COOH) C20:3 | 45.92 | 284 | 7.10E-05 | 0.234 | 5.45% |
| SM (OH) C22:0 | 17.06 | 284 | 7.73E-05 | 0.232 | 5.40% |
| SM (OH) C22:2 | 31.89 | 284 | 8.05E-05 | 0.232 | 5.37% |
| SM (OH) C24:0 | 11.80 | 208 | 1.14E-04 | 0.264 | 6.99% |
| SM (OH,COOH) C24:1 | 22.98 | 284 | 1.88E-04 | 0.220 | 4.83% |
| SM (OH) C20:1 | 16.74 | 284 | 3.04E-04 | 0.213 | 4.53% |
| SM (OH) C26:0 | 30.79 | 284 | 7.70E-04 | 0.198 | 3.94% |
| SM (OH) C20:0 | 25.82 | 284 | 1.23E-03 | 0.191 | 3.64% |
| PE a C10:0 | 4.16 | 284 | 1.26E-03 | 0.191 | 3.63% |
| PE e (COOH) C12:1* | 42.07 | 284 | 1.32E-03 | 0.190 | 3.60% |
| SM (COOH) 18:0 | 21.85 | 221 | 1.55E-03 | 0.212 | 4.48% |
| SM (OH) C28:0 | 59.33 | 284 | 2.44E-03 | 0.179 | 3.21% |
| SM (OH,COOH) C20:0 | 13.60 | 132 | 2.79E-03 | 0.258 | 6.67% |
| PC aa C34:4 | 3.25 | 284 | 3.06E-03 | 0.175 | 3.07% |
| SM (OH,COOH) C20:1 | 10.98 | 69 | 4.61E-03 | 0.337 | 11.37% |
| SM (OH) C28:1 | 24.76 | 284 | 6.80E-03 | 0.160 | 2.57% |
| PG aa (OH, COOH) C20:2 | 17.35 | 63 | 7.45E-03 | 0.334 | 11.16% |
| PE e C16:3* | 4.72 | 139 | 1.59E-02 | 0.204 | 4.17% |
| PC aa C36:5 | 47.53 | 284 | 1.93E-02 | 0.139 | 1.93% |
| PC ae C36:0 | 3.42 | 284 | 2.13E-02 | 0.137 | 1.87% |
| PG a (OH, COOH) C14:3 | 290.41 | 76 | 2.24E-02 | 0.262 | 6.85% |
| PE aa (OH, COOH) C24:2 | 4.58 | 63 | 2.52E-02 | -0.282 | 7.94% |
| SM (OH,COOH) C18:0 | 35.99 | 63 | 3.07E-02 | 0.273 | 7.43% |
| PE e C16:3 | 5.27 | 63 | 3.10E-02 | 0.272 | 7.40% |
| PC aa C38:4 | 209.05 | 284 | 3.24E-02 | 0.127 | 1.61% |
| PC a C20:4 | 5.09 | 284 | 3.32E-02 | 0.126 | 1.60% |
| GalCer sulf C18:0 | 31.85 | 61 | 3.81E-02 | 0.266 | 7.09% |
| PC aa C36:4 | 399.41 | 284 | 3.92E-02 | 0.122 | 1.50% |
| PC aa C30:2 | 8.99 | 284 | 3.96E-02 | 0.122 | 1.49% |
| SM (COOH) 18:1 | 15.29 | 152 | 4.02E-02 | 0.167 | 2.78% |
| PIP aa C30:2 | 2.92 | 76 | 4.21E-02 | 0.234 | 5.46% |
| PC a C26:1 | 0.91 | 63 | 4.93E-02 | 0.249 | 6.19% |
| PC aa C38:3 | 123.00 | 284 | 4.97E-02 | 0.117 | 1.36% |
