# Supplementary material for: Genetics Meets Metabolomics: A Genome-Wide Association Study of Metabolite Profiles in Human Serum
Source: PLoS Genet. 2008 Nov 28;4(11):e1000282. doi: 10.1371/journal.pgen.1000282 (PMC2581785; doi:10.1371/journal.pgen.1000282)
Supplement: Table S5 — Associations of rs992037 (PARK2) with metabolic traits. Metabolites associated (p<0.05) with genotype rs992037 (PARK2) in the additive genetic model (see Table S2 for legend). (0.08 MB DOC) [file pgen.1000282.s006.doc]

| **metabolite** | **mean** | **ncases** | **p-value** | **estimate** | **explained variance** |
| --- | --- | --- | --- | --- | --- |
| LYS | 215.17 | 284 | 1.20E-07 | 0.308 | 9.48% |
| PC aa C38:1* | 18.72 | 284 | 3.39E-03 | 0.173 | 3.00% |
| GLY | 162.50 | 284 | 5.28E-03 | 0.165 | 2.73% |
| HIS | 76.52 | 284 | 6.17E-03 | 0.162 | 2.63% |
| C16:1 | 0.04 | 284 | 6.19E-03 | -0.162 | 2.63% |
| GLU | 129.77 | 284 | 7.02E-03 | -0.160 | 2.55% |
| C6 | 0.09 | 284 | 8.41E-03 | -0.156 | 2.44% |
| PC aa C42:6 | 1.27 | 63 | 9.04E-03 | -0.326 | 10.65% |
| SM (OH,COOH) C20:2 | 64.74 | 284 | 9.06E-03 | 0.155 | 2.39% |
| C14:1 | 0.13 | 284 | 1.03E-02 | -0.152 | 2.31% |
| C18:2-OH | 0.05 | 284 | 1.18E-02 | -0.149 | 2.23% |
| Docosahexaonic acid | 4.14 | 283 | 1.32E-02 | -0.147 | 2.16% |
| SM (OH,COOH) C24:0 | 20.93 | 284 | 1.49E-02 | 0.144 | 2.09% |
| PC ae C22:8 | 11.53 | 76 | 1.72E-02 | 0.273 | 7.43% |
| ARG | 115.97 | 284 | 2.01E-02 | 0.138 | 1.90% |
| ORN | 57.03 | 284 | 2.27E-02 | 0.135 | 1.83% |
| PI a (OH, COOH) C14:2 | 3.74 | 76 | 2.37E-02 | -0.259 | 6.73% |
| Methionine Sulfonate | 2.39 | 284 | 2.45E-02 | 0.133 | 1.78% |
| C2 | 8.60 | 284 | 2.59E-02 | -0.132 | 1.75% |
| PA aa C42:5 | 84.31 | 284 | 2.62E-02 | -0.132 | 1.74% |
| C18:1 | 0.16 | 284 | 2.99E-02 | -0.129 | 1.66% |
| C12:1 | 0.12 | 284 | 3.06E-02 | -0.128 | 1.65% |
| PE aa C40:5 | 1.57 | 132 | 3.76E-02 | -0.181 | 3.28% |
| SM (OH,COOH) C18:1 | 19.27 | 284 | 3.76E-02 | 0.123 | 1.52% |
| SM (OH,COOH) C14:1 | 2.16 | 221 | 3.91E-02 | -0.139 | 1.93% |
| PC a C22:5 | 0.81 | 63 | 4.13E-02 | -0.258 | 6.65% |
| PC ae C38:0* | 5.24 | 284 | 4.35E-02 | -0.120 | 1.44% |
| C10 | 0.32 | 284 | 4.35E-02 | -0.120 | 1.44% |
| SM C28:3 | 2.59 | 284 | 4.87E-02 | 0.117 | 1.37% |
| SM (OH,COOH) C18:2 | 92.45 | 284 | 4.95E-02 | 0.117 | 1.36% |
