# Supplementary material for: Genetics Meets Metabolomics: A Genome-Wide Association Study of Metabolite Profiles in Human Serum
Source: PLoS Genet. 2008 Nov 28;4(11):e1000282. doi: 10.1371/journal.pgen.1000282 (PMC2581785; doi:10.1371/journal.pgen.1000282)
Supplement: Table S6 — Associations of rs992037 (PARK2) with metabolite concentration ratios. Selected metabolite concentration ratios associated (p<0.05) with genotype rs992037 (PARK2) in the additive genetic model (see Table S2 for legend; ncases = 284). The improvement of the p-value of association when using metabolite concentration ratios is calculated based on the following formula: min(p[C_enumerator], p[C_nominator]) / p[C_enumerator / C_nominator], where C_ is a metabolite concentration and p[.] the corresponding p-value of association. (0.06 MB DOC) [file pgen.1000282.s007.doc]

| **enumerator** | **nominator** | **Mean** | **p-value** | **estimate** | **improvement of p-value** |
| --- | --- | --- | --- | --- | --- |
| ARG | GLU | 0.927 | 4.73E-06 | 0.268 | 1484.4 |
| MET | GLU | 0.333 | 5.75E-06 | 0.265 | 1221.3 |
| LYS | GLU | 1.773 | 6.79E-06 | 0.263 | 0.018 |
| GLY | GLU | 1.305 | 1.07E-05 | 0.258 | 491.1 |
| ORN | GLU | 0.455 | 1.16E-05 | 0.257 | 604.8 |
| HIS | GLU | 0.613 | 2.37E-05 | 0.248 | 260.0 |
| TRP | GLU | 0.629 | 3.20E-05 | 0.244 | 219.2 |
| THR | GLU | 0.540 | 5.86E-05 | 0.236 | 119.8 |
| PHE | GLU | 0.575 | 5.94E-05 | 0.236 | 118.1 |
| MET.SULF | GLU | 0.019 | 1.21E-04 | 0.226 | 58.1 |
| TYR | GLU | 0.514 | 2.60E-04 | 0.215 | 27.0 |
| ALA | GLU | 2.248 | 4.45E-04 | 0.207 | 15.8 |
| CIT | GLU | 0.152 | 1.28E-03 | 0.190 | 5.5 |
